# Supplementary material for: Salience memories formed by value, novelty and aversiveness jointly shape object responses in the prefrontal cortex and basal ganglia
Source: Nat Commun. 2022 Oct 25;13:6338. doi: 10.1038/s41467-022-33514-3 (PMC9596424; doi:10.1038/s41467-022-33514-3)
Supplement: Supplementary file 1 — Supplementary Information [file 41467_2022_33514_MOESM1_ESM.pdf]

Supplemental Information

Salience Memories Formed by Value, Novelty and Aversiveness Jointly Shape Object Responses in the Prefrontal Cortex and Basal Ganglia

Ali Ghazizadeh[1*] and Okihide Hikosaka[2,3]

[1]Bio-intelligence Research Unit, Electrical Engineering Department, Sharif University of Technology, Tehran 11365-11155, Iran

[2]Laboratory of Sensorimotor Research, National Eye Institute, NIH, Bethesda, MD 20892, USA

[3]National Institute on Drug Abuse, NIH, Baltimore, MD 21224, USA

* Corresponding author: alieghazizadeh@gmail.com

Supplementary Figure 1

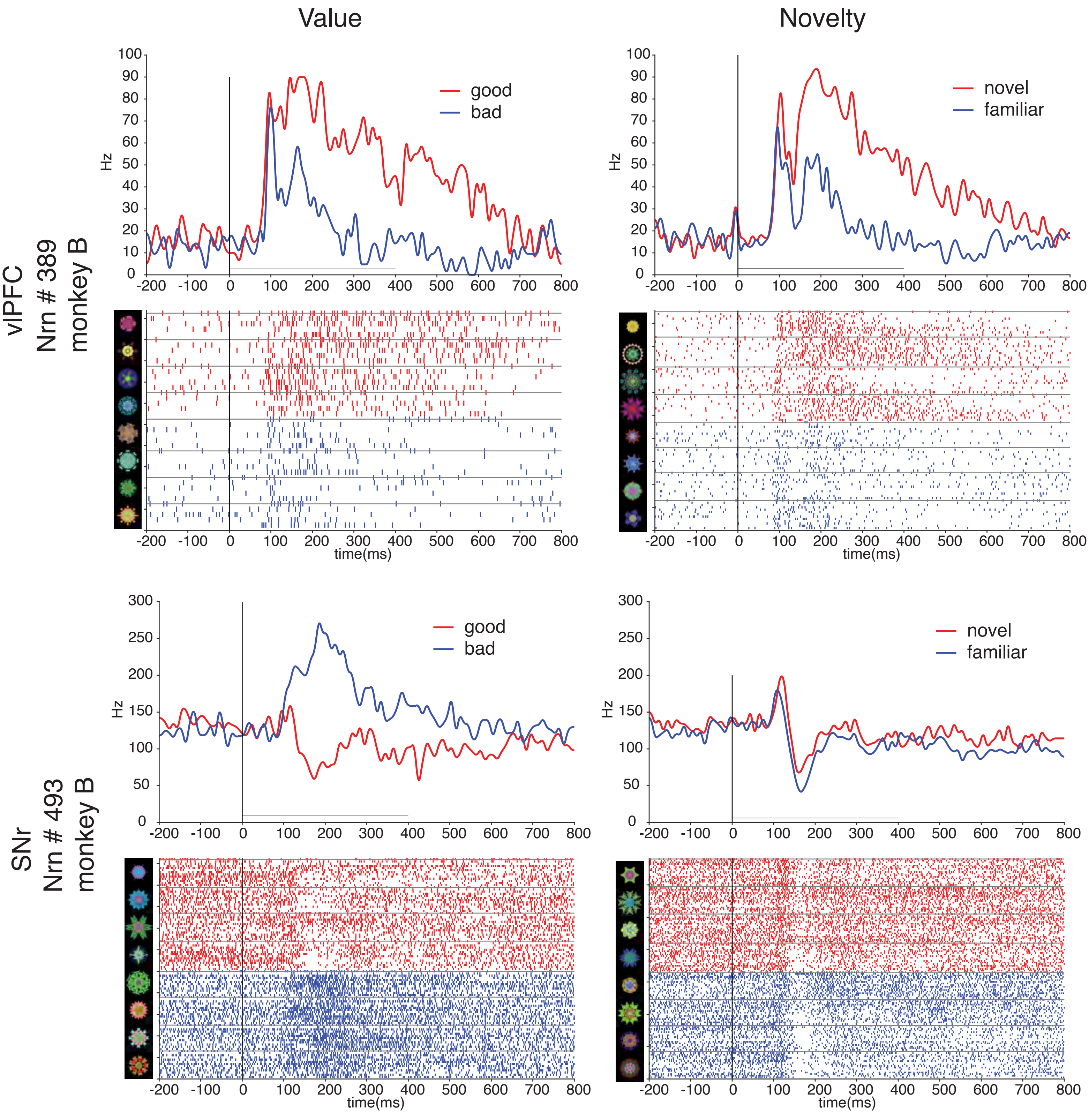

Supplementary Figure 1. Example passive viewing responses to objects in value and novelty domains in vlPFC and cdlSNr.
Example neurons recorded with both a good/bad set and a novel/familiar set in vlPFC (top row) and in cdlSNr (bottom row). In each row the top part shows average peristimulus time histograms (PSTH) of firing to two object categories and the bottom part shows raster plot of firing sorted for each fractal object. Actual fractals used are shown to the left and grouped into good/bad and novel/familiar. Horizontal line in the PSTH indicates object on duration (400ms)

Supplementary Figure 2

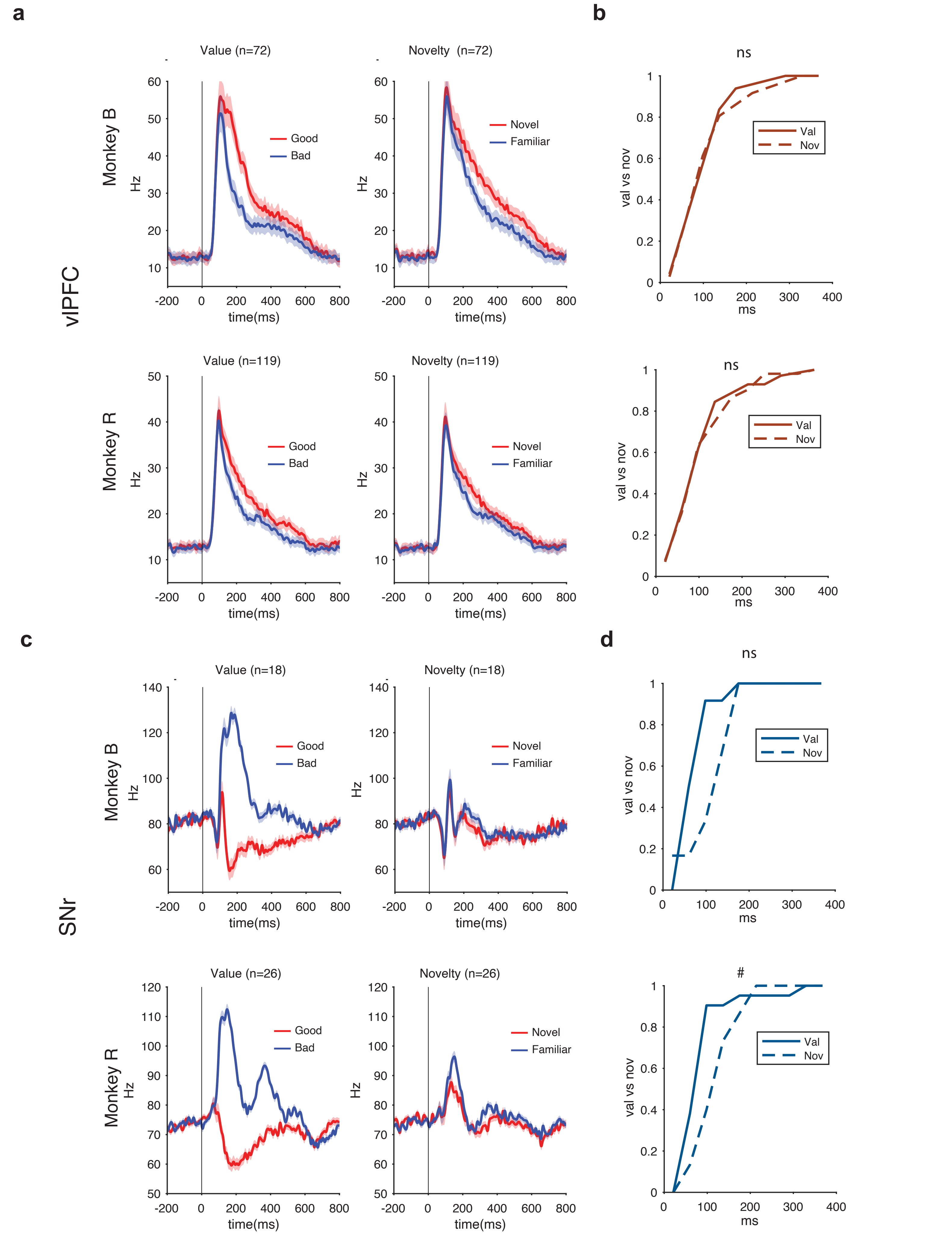

Supplementary Figure 2. Responses of vlPFC and cdlSNr neurons and their latencies to good/bad and novel/familiar objects separately for each monkey.

a) Population average peristimulus time histogram (PSTH) to good vs bad objects and to novel vs familiar objects in vlPFC in each monkey. Color-patch indicates s.e.m. here and throughout. b) Cumulative distribution of value signal and novelty signal onsets across neurons in vlPFC for each monkey (monkey B: t_{83}=-0.5, p=0.5, monkey R: t_{121}=-0.2, p=0.7). c) Same as A but for SNr in each monkey. D) Same as B but for cdlSNr in each monkey (monkey B: t_{16}=-1, p=0.3, monkey R: t_{34}=-1.9, p=0.06). Note, despite large number of recorded SNr neurons, since novelty coding exists in a small subset of them, the degree of freedom for comparison of value and novelty onset in SNr is low and thus despite clear trend of novelty coding being later than value coding in SNr in both monkeys, it does not reach significance in individual monkeys. The combined data is however significant as shown in Fig 1F.

Supplementary Figure 3

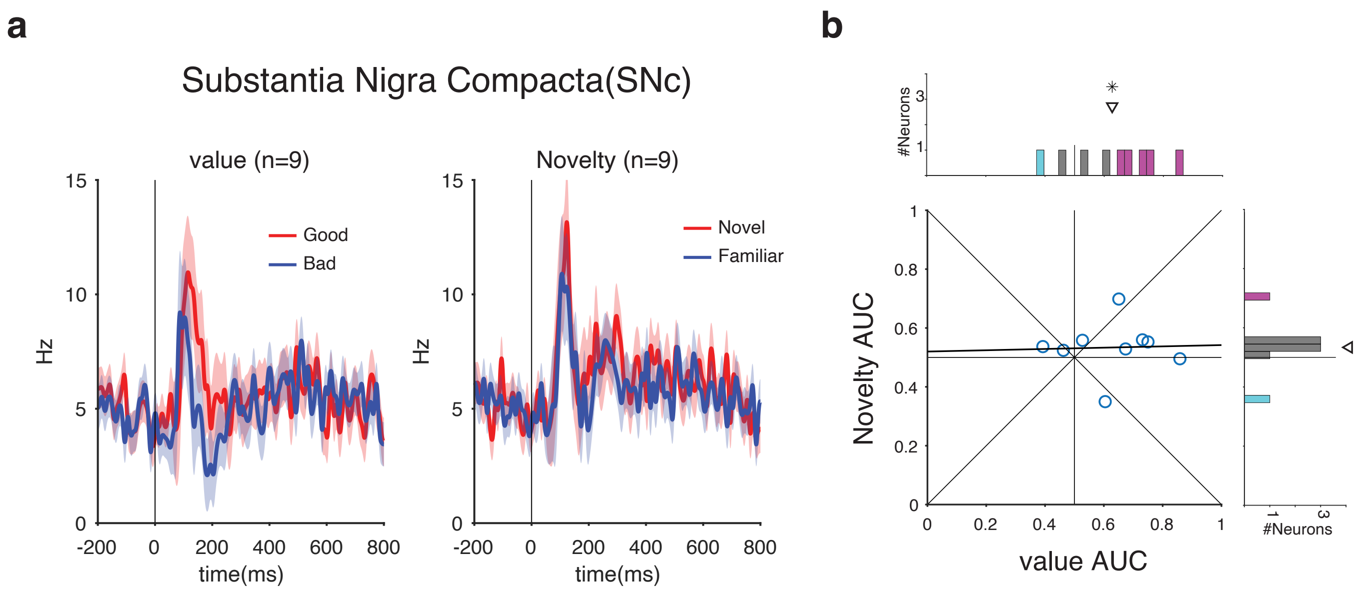

Supplementary Figure 3. Responses of putative dopamine neurons to good/bad and novel/familiar objects. a) Same format as Figure 1C. b) Same format as Figure 1G. Source data are provided as a Source Data file.

Supplementary Figure 4

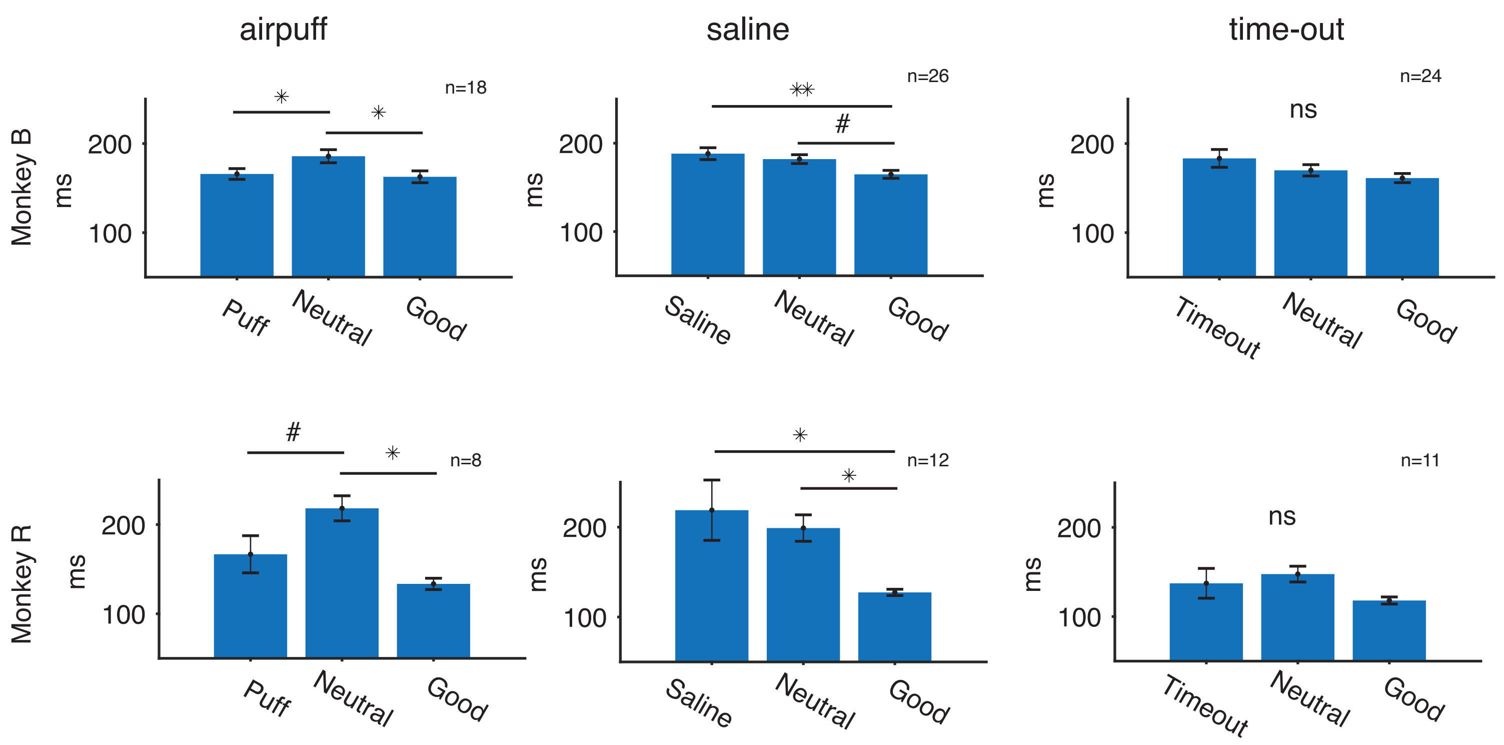

Supplementary Figure 4. Saccade reaction time to aversive, neutral and good objects during Pavlovian task

Reaction time (RT) to look at aversive, neutral and good objects after their appearance in the periphery during force trials in Pavlovian task in monkey B for airpuff set ($F_{2,51}$=3.49, p=3e-2), salience set ($F_{2,75}$=4.89, p=1e-2) and time-out set ($F_{2,68}$=2.18, p=0.12) and in monkey R for for airpuff set ($F_{2,21}$=6.88, p=2e-3), salience set ($F_{2,30}$=6.88, p=3e-3) and time-out set ($F_{2,30}$=1.8, p=0.18). In both monkeys RT to airpuff object was faster than neutral objects consistent with its positive salience. RT to saline and time-out objects were not different from neutral object consistent with their lack of salience. For time-out objects differences were not significant but had a similar trend to saline objects. Note that in the Pavlovian task monkey's action (to look or not to look at fractals) had no consequence for outcome delivery. post-hoc hsd comparisons are marked. Data are presented as mean values +/- SEM.**p<0.01, * p<0.05, #p<0.08. Source data are provided as a Source Data file.

Supplementary Figure 5

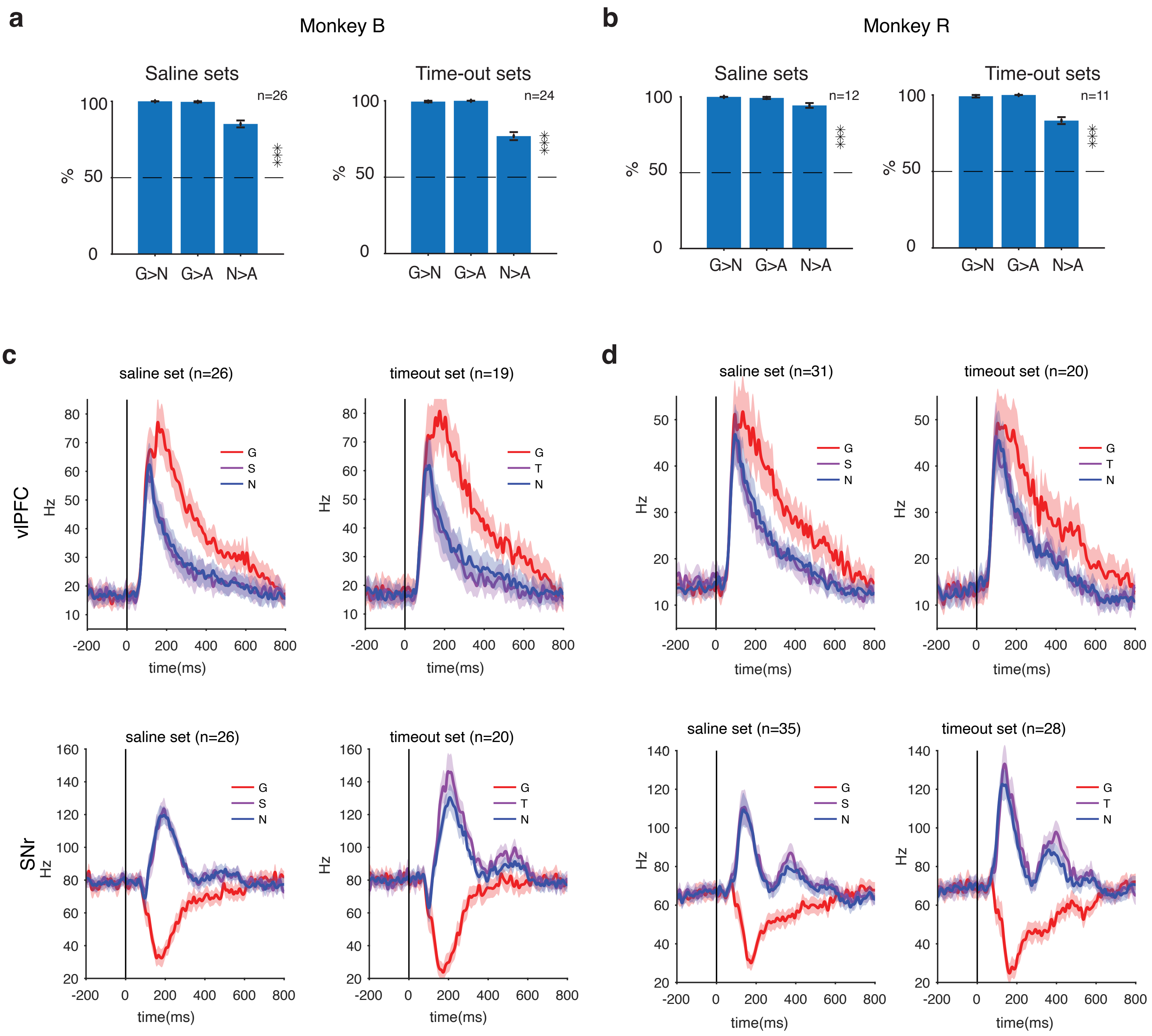

Supplementary Figure 5. Choice performance and neuronal responses of vlPFC and cdlSNr to saline and time-out sets separately for each monkey.
a-b) Same format as Figure 4B. (Monkey B: saline, N>A t_{11}=28, p=1e-11; time-out, N>A t_{10}=14, p=4e-8. Monkey R: saline, N>A t_{15}=25, p=2e-14; time-out, N>A t_{23}=10, p=7e-10.) C-D) Same format as Figure 4D. Data in A and B are presented as mean values +/- SEM. Source data are provided as a Source Data file.

Supplementary Figure 6

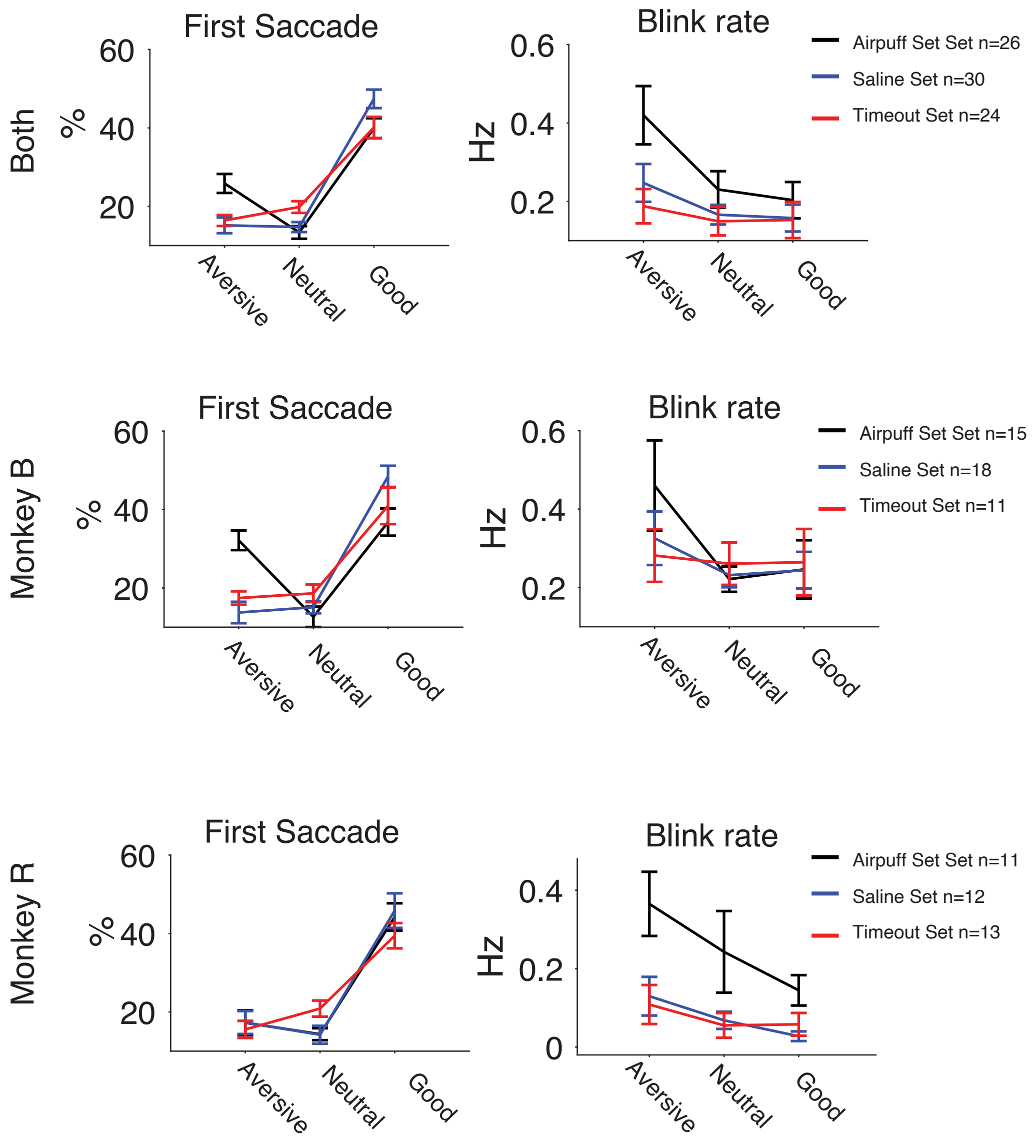

Supplementary Figure 6. First saccade and blink rates during free viewing for airpuff, salience and time-out sets for both monkeys and separately for each monkey.
Free viewing gaze bias using first saccade following display onset toward aversive, neutral and good objects and the blinking rate during fixating the object in free viewing for airpuff, saline and time-out sets. The number of sessions in each set type is noted in the figure. Data are presented as mean values +/- SEM. Detailed stats are shown in Supplementary Table 1.

Supplementary Figure 7

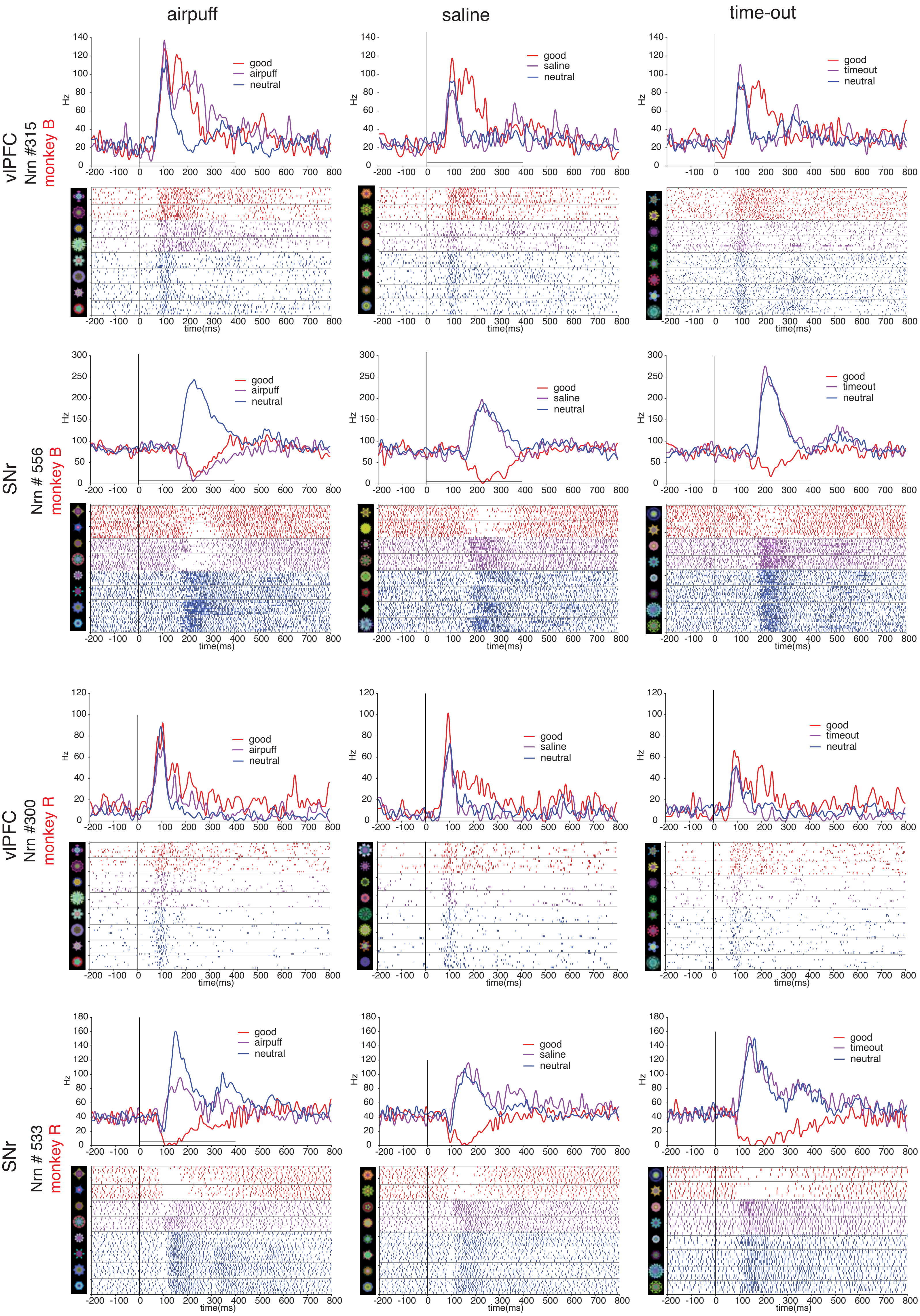

Supplementary Figure 7. Example passive viewing responses to objects to airpuff, saline and time-out sets in vlPFC and cdlSNr.
Example neurons recorded with all three aversive set types in vlPFC and cdlSNr in each monkey. Same format as Supplementary Figure 1.

Supplementary Figure 8

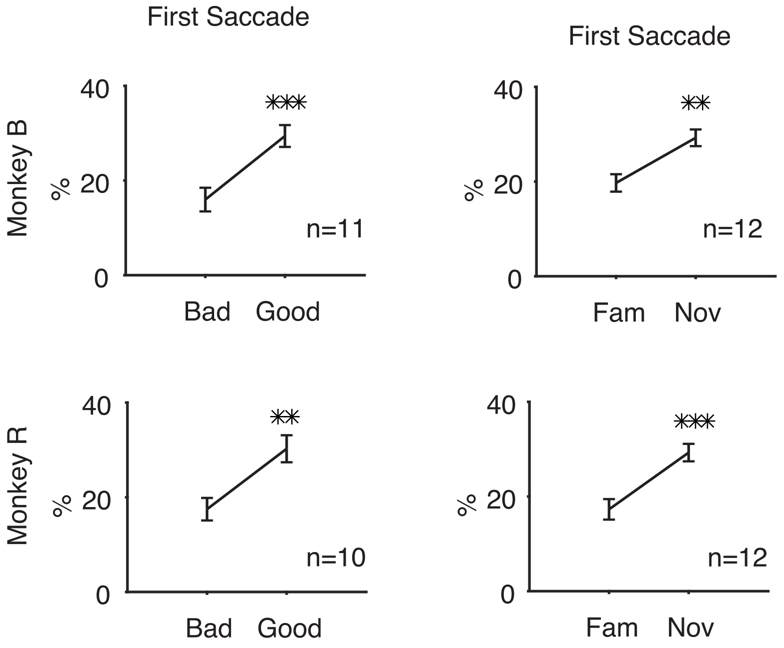

Supplementary Figure 8. First saccade during free viewing for good/bad and novel/familiar separately for each monkey.
Free viewing gaze bias using first saccade following display onset toward good vs bad (Monkey B, t_{20}=3.9 p=8e-4, Monkey R, t_{18}=3.4 p=2e-3) and novel vs familiar objects (Monkey B, t_{22}=3.7 p=1e-3, Monkey R, t_{22}=4 p=3e-4). Source data are provided as a Source Data file.

Supplementary Figure 9

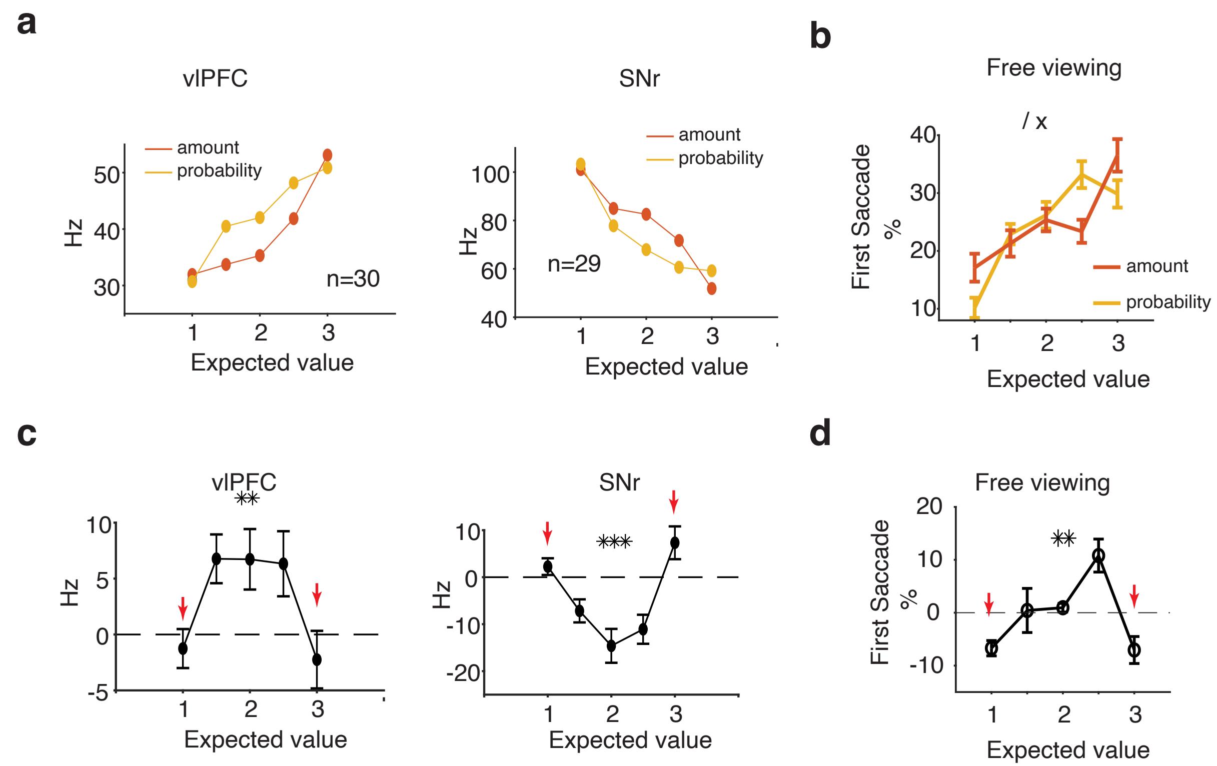

Supplementary Figure 9. Graded response of vlPFC and cdlSNr to objects with graded increase in reward amount and probability and corresponding graded change in object salience in free viewing
a) Average firing of neurons to objects from amount, probability sets as a function of expected value in vlPFC (top) and cdlSNr (bottom). b) First saccade bias for objects in probability and amount sets as a function of their expected value (first saccade $F_{4,395}>4.8$ $p<8e-4$ main effect of value and interaction). Total of 38 amount and 43 probability sessions, / and X indicate main effect of value and interaction, respectively. c) Average firing difference for objects with the same expected value from probability sets compared to amount sets in vlPFC (top, $F_{4,145}=3.5$, p=9e-3) and cdlSNr (bottom, $F_{4,140}=9.6$, p=6e-7). d) Difference in gaze bias for probability sets compared to amount sets (first saccade $F_{4,15}=7.2$ p=1e-3). The red arrows annotate the difference in response at lowest and highest values to amount and probability objects despite matching value and lack of uncertainty. This effect can also be seen if attentional bias to amount and value are subtracted in free viewing but has no easy interpretation in predictions made within value or risk frameworks. Data in B, C and D are presented as mean values +/- SEM. [adapted and reproduced from Ghazizadeh & Hikosaka 2021 with permission from Science Advances]

Supplementary Figure 10

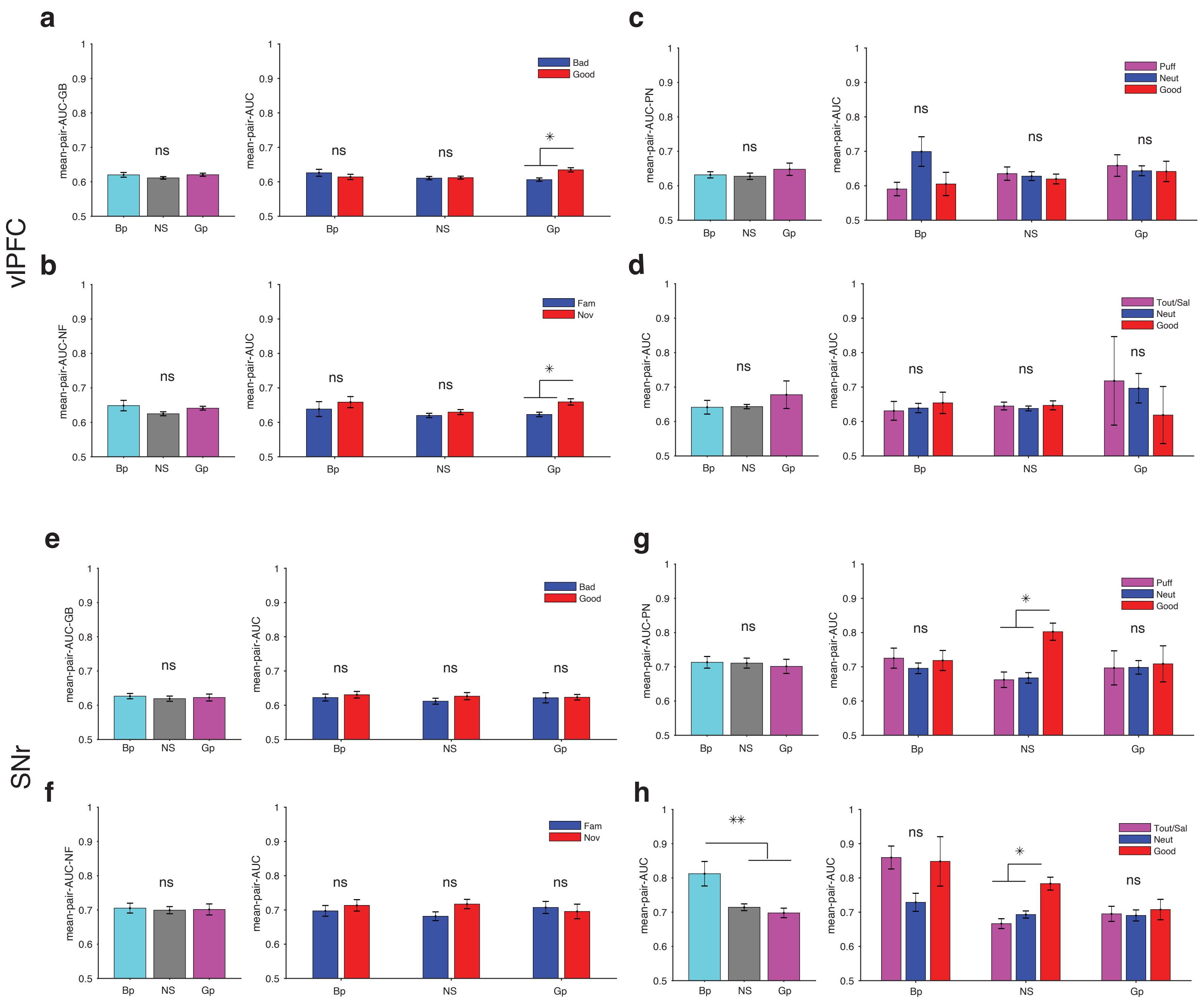

Supplementary Figure 10. Within category object discrimination using pairwise AUC among objects in vlPFC and cdlSNr separately for Gp, NS and Bp neurons.

a) vlPFC object discrimination for good/bad objects across neuron types (left; $F_{2,346}$=1.4 p=0.2) and separately for good and bad objects across neuron types (right; $F_{1,692}$=1.1, p=0.2, interaction $F_{2,692}$=5.3, p=4e-3). b) vlPFC object discrimination for novel/familiar objects across neuron types (left; $F_{2,189}$=2.6 p=0.07) and separately for novel and familiar objects across neuron types (right; $F_{1,378}$=5.4, p=0.02, interaction $F_{2,378}$=1.6, p=0.1). c) vlPFC object discrimination for airpuff/neutral /good objects across neuron types (left; $F_{2,56}$=0.6 p=0.5) and separately for airpuff, neutral and good objects across neuron types (right; $F_{2,168}$=1.2, p=0.27, interaction $F_{4,168}$=1, p=0.3). d) vlPFC object discrimination for saline or time-out/neutral /good objects across neuron types (left; $F_{2,93}$=0.5 p=0.5) and separately for saline/timeout, neutral and good objects across neuron types (right; $F_{2,279}$=0.3, p=0.6, interaction $F_{4,279}$=0.5, p=0.6). e) cdlSNr object discrimination for good/bad objects across neuron types (left; $F_{2,113}$=0.2 p=0.7) and separately for good and bad objects across neuron types (right; $F_{1,226}$=0.5, p=0.4 , interaction $F_{2,226}$=0.1, p=0.8). f) cdlSNr object discrimination for novel/familiar objects across neuron types (left; $F_{2,80}$=0.06 p=0.9) and separately for novel and familiar objects across neuron types (right; $F_{1,160}$=0.9, p=0.3, interaction $F_{2,160}$=0.9, p=0.4). g) cdlSNr object discrimination for airpuff/neutral /good objects across neuron types (left; $F_{2,62}$=0.05 p=0.9) and separately for airpuff, neutral and good objects across neuron types (right; $F_{2,186}$=2.5, p=0.08, interaction $F_{4,186}$=3, p=0.01). h) cdlSNr object discrimination for saline/time-out/neutral /good objects across neuron types (left; $F_{2,106}$=5.5 p=5e-3) and separately for saline/timeout, neutral and good objects across neuron types (right; $F_{2,318}$=4.1, p=0.01, interaction $F_{4,318}$=3, p=0.01). Given similar results for saline and time-out objects, results are combined for the two set types. Data in a-h are presented as mean values +/- SEM.

Supplementary Figure 11

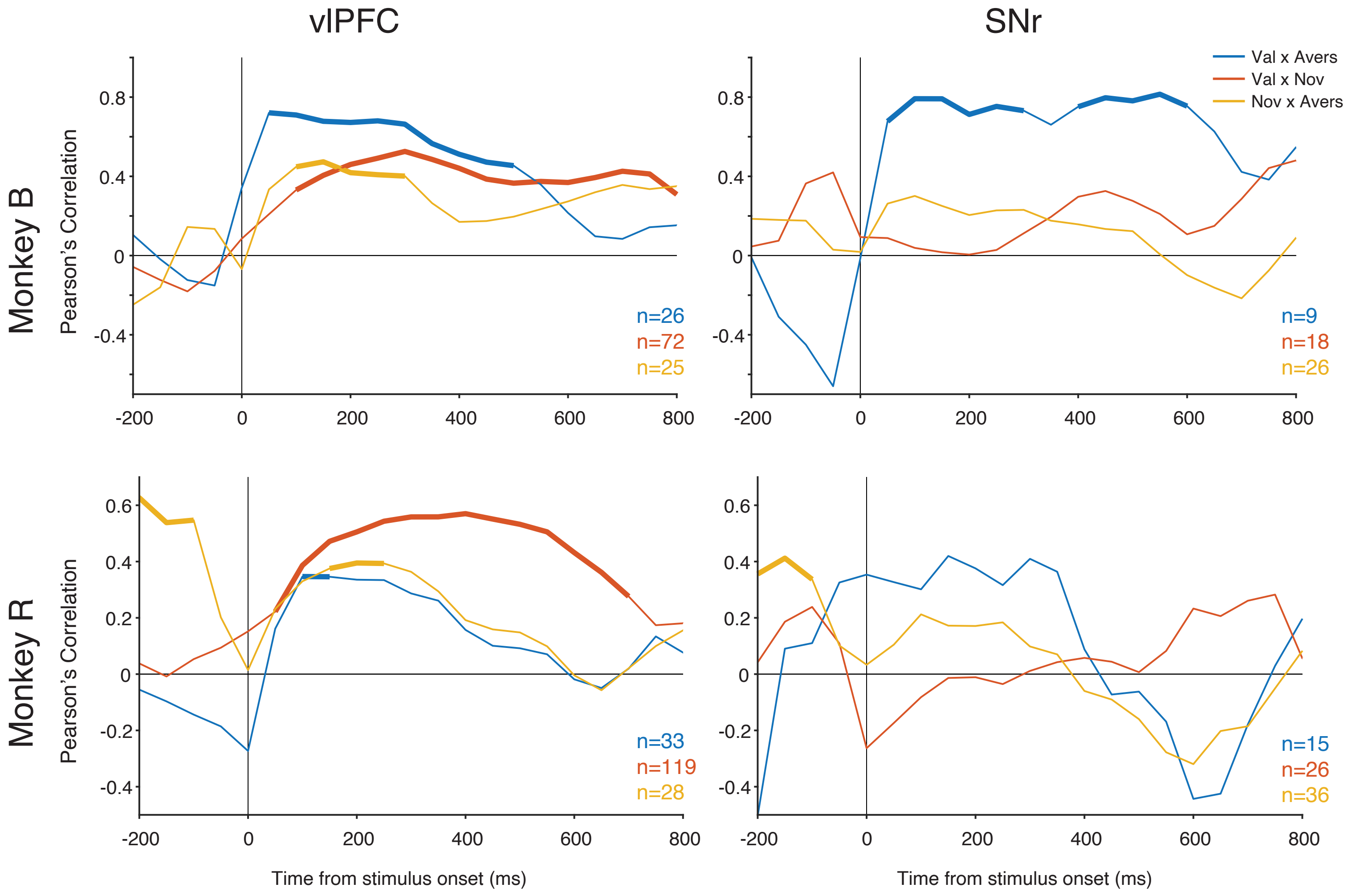

Supplementary Figure 11. Sliding pairwise correlation across value, novelty and aversive (airpuff) dimension for vlPFC and cdlSNr.
Same format as Figure 6 but separately for each monkey.

Supplementary Figure 12

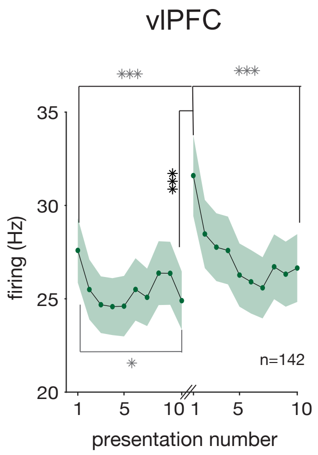

Supplementary Figure 12. Sensitivity of vlPFC but not cdlSNr to object recency. Population average firing in two consequent blocks of passive viewing done with two different object sets showing recovery of response suppression in vlPFC (top, $F_{9,2547}$=10 p=2e-9 main effect of trial, last trial of first block vs first trial of second block t141=4.9 p=2e-6, first vs last trial of first first block t_{141}=2.3 p=0.01, first vs last trial of second block t_{141}=4.4 p=2e-5, first trial of first vs second block t141=3.6 p=3e-4) but not in cdlSNr (bottom, $F_{9,801}$=1.3 p=0.18 main effect of trial, last trial of first block vs first trial of second block t_{44}=0.44 p=0.9, other pairwise tests shown abs(t_{141})<0.9 p>0.4). Data are presented as mean values +/- SEM. [reproduced with permission from Science Advances]

Supplementary Figure 13

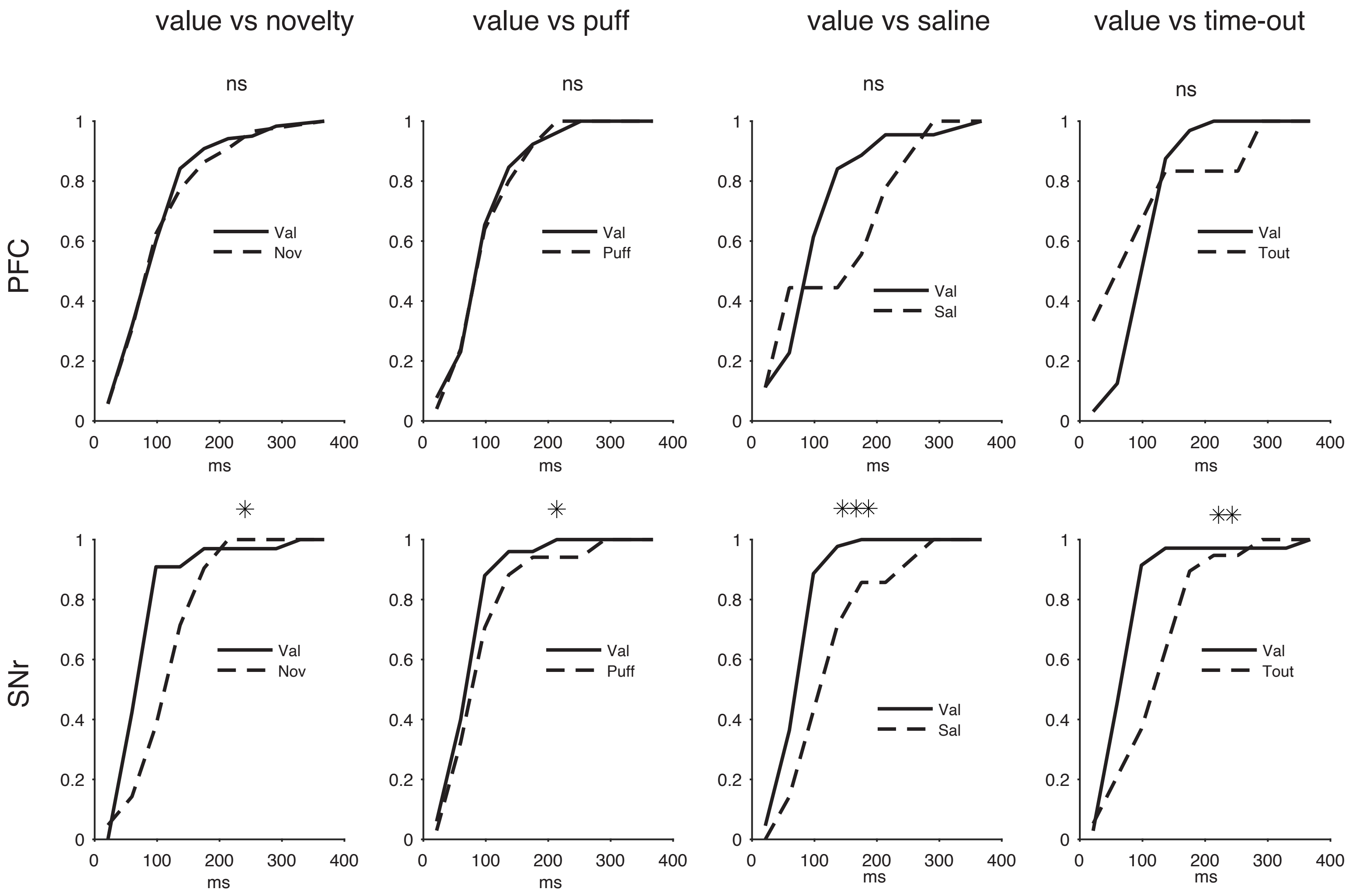

Supplementary Figure 13. Cumulative onset distributions comparison for value vs novelty, value vs airpuff, value vs saline and value vs time-out signals within vlPFC and cdlSNr neurons. Novelty vs value signal onsets from neurons that have both GB and NF sets in vlPFC (average 119 vs 113 t_{206}=-0.5, p=0.5) in cdlSNr (average 128 vs 94 ms, t_{52}=-2.3, p=0.02). Airpuff vs value signal onsets from neurons recorded with airpuff sets (value signal comes from good minus neutral and airpuff signal comes from airpuff minus neutral) in vlPFC (average 116 vs 110, t_{75}=-0.5, p=0.6) and in cdlSNr (average 107 vs 86ms, t_{82}=-2, p=0.04). Saline vs value signal onsets from neurons recorded with saline sets (value signal comes from good minus neutral and saline signal comes from saline minus neutral) in vlPFC (average 146 vs 116ms t_{51}=-1.1, p=0.2) and in cdlSNr (average 132 vs 88ms, t_{56}=-3.5, p=7e-4). Timeout vs value signal onsets from neurons recorded with time-out sets (value signal comes from good minus neutral and time-out signal comes from time-out minus neutral) in vlPFC (average 100 vs 114ms t_{36}=0.6, p=0.5) and in cdlSNr (average 135 vs 91ms, t_{52}=-2.7, p=8e-3).

Supplementary Figure 14

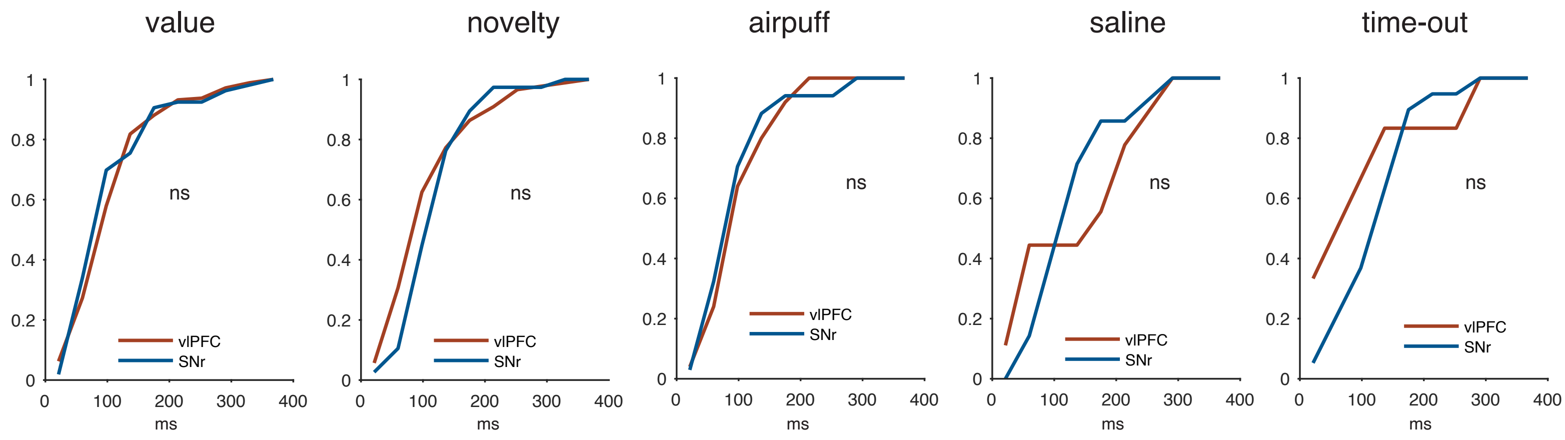

Supplementary Figure 14. Cumulative onset distributions comparison for value, novelty, airpuff, saline and time-out signals between vlPFC and cdlSNr neurons.

Value signal onsets between neurons recorded with GB sets in vlPFC and cdlSNr (average 120 vs 117 ms, t_{227}=0.2, p=0.8). Novelty signal onsets between neurons recorded with NF sets in vlPFC and cdlSNr (average 119 vs 131 ms, t_{124}=-0.9, p=0.3). Airpuff signal onsets between neurons recorded with airpuff sets in vlPFC and cdlSNr (average 116 vs 107 ms, t_{57}=0.6, p=0.4). Saline signal onsets between neurons recorded with saline sets in vlPFC and cdlSNr (average 146 vs 132 ms, t_{21}=0.4, p=0.6). Time-out signal onsets between neurons recorded with time-out sets in vlPFC and cdlSNr (average 100 vs 135 ms, t_{23}=-1, p=0.2).

Supplementary Table 1. Detailed stats for data shown in Supplementary Fig 6.

	Aversive set	Measure	ANOVA F	ANOVA P	A vs N	A vs G	N vs G
Both	airpuff	First saccade percentage	F2,74=35	1E-11	0.0005	0.0001	<1e-4
		Blinking rate	F2,74=4.2	0.018	0.057	0.025	0.94
	saline	First saccade percentage	F2,86=92	3E-22	0.97	<1e-4	<1e-4
		Blinking rate	F2,86=2.2	0.11	0.19	0.13	0.98
	Timeout	First saccade percentage	F2,68=42	1E-12	0.44	<1e-4	<1e-4
		Blinking rate	F2,68=0.32	0.72	0.75	0.78	0.99
Bongo	airpuff	First saccade percentage	F2,42=19.7	9.00E-07	0.0001	0.5	<1e-4
		Blinking rate	F2,42=2.5	8.00E-02	0.1	0.16	0.9
	saline	First saccade percentage	F2,51=67	4.00E-15	0.9	<1e-4	<1e-4
		Blinking rate	F2,51=1	3.70E-01	0.4	0.5	0.98
	Timeout	First saccade percentage	F2,30=1	8.00E-06	0.9	<1e-4	0.0001
		Blinking rate	F2,30=0.02	9.70E-01	0.97	0.98	0.99
Rheno	airpuff	First saccade percentage	F2,30=33	2.00E-08	0.75	<1e-4	<1e-4
		Blinking rate	F2,30=1.9	1.60E-01	0.52	0.14	0.66
	saline	First saccade percentage	F2,33=27	9.00E-08	0.78	<1e-4	<1e-4
		Blinking rate	F2,33=2.5	9.00E-02	0.37	0.07	0.64
	Timeout	First saccade percentage	F2,36=24	2.00E-07	0.31	<1e-4	<1e-4
		Blinking rate	F2,36=0.6	5.00E-01	0.58	0.61	0.99
