# Supplementary material for: Salience memories formed by value, novelty and aversiveness jointly shape object responses in the prefrontal cortex and basal ganglia
Source: Nat Commun. 2022 Oct 25;13:6338. doi: 10.1038/s41467-022-33514-3 (PMC9596424; doi:10.1038/s41467-022-33514-3)
Supplement: Supplementary file 3 — Description of Additional Supplementary Files [file 41467_2022_33514_MOESM3_ESM.pdf]

Description of Additional Supplementary Files

File name: Supplementary Data 1

Description: This data contains the spiking times for each trial of each neuron time-locked to stimulus onset in value, novelty and aversive conditions. Explanation of the data structure is provided in the accompanying Readme file.
