# Supplementary material for: Salience memories formed by value, novelty and aversiveness jointly shape object responses in the prefrontal cortex and basal ganglia
Source: Nat Commun. 2022 Oct 25;13:6338. doi: 10.1038/s41467-022-33514-3 (PMC9596424; doi:10.1038/s41467-022-33514-3)
Supplement: Supplementary file 4 — Supplementary Data 1 [file 41467_2022_33514_MOESM4_ESM.zip › Readme.rtf]

The data in ‘SalMem_PFC_SNr.mat’PSR_substack [nx1 Cell] :Contains the spike times for all neurons in all conditions time locked to object onset in passive viewing. Each row is one trialNrnSubjRegTypSet0ObjtypObj_plex [nx1 vector]: Each row is one trial matching the rows in PSR_substack. It has information about each trial multiplexed into : Neuron number, Subject number [1:Monkey B, 2:Monkey R], Region [1:vlPFC, 2: SNr, 3: DA], Typ [1 : GB, 2: NF, 5:Puff , 6: Saliine , 7:Timeout], Set number [4 digits],… ObjTyp [ for GB and NF sets 1: Good/Novel 2: Bad/Familiar, for aversive sets 1:Good 2:Aversive, 3: Neutral], Obj number [for GB and NF sets1:4 Good/Novel, 5:8 Bad/Familiar, for aversive sets 1:2 Good, 3:4 Aversive, 5:8 Neutral]The NrnSubjRegTypSet0ObjtypObj_plex can be used to choose appropriate rows in PSR_substack for making peristimulus time histograms (PSTH). 
