# Supplementary material for: Salience memories formed by value, novelty and aversiveness jointly shape object responses in the prefrontal cortex and basal ganglia
Source: Nat Commun. 2022 Oct 25;13:6338. doi: 10.1038/s41467-022-33514-3 (PMC9596424; doi:10.1038/s41467-022-33514-3)
Supplement: Supplementary file 5 — Reporting Summary [file 41467_2022_33514_MOESM5_ESM.pdf]

Corresponding author(s): Ali Ghazizadeh

Last updated by author(s): Dec 8, 2021

Reporting Summary

Nature Portfolio wishes to improve the reproducibility of the work that we publish. This form provides structure for consistency and transparency in reporting. For further information on Nature Portfolio policies, see our Editorial Policies and the Editorial Policy Checklist.

Statistics

For all statistical analyses, confirm that the following items are present in the figure legend, table legend, main text, or Methods section.

n/a	Confirmed	
☐	☒	The exact sample size (*n*) for each experimental group/condition, given as a discrete number and unit of measurement
☐	☒	A statement on whether measurements were taken from distinct samples or whether the same sample was measured repeatedly
☐	☒	The statistical test(s) used AND whether they are one- or two-sided<br>*Only common tests should be described solely by name; describe more complex techniques in the Methods section.*
☐	☒	A description of all covariates tested
☐	☒	A description of any assumptions or corrections, such as tests of normality and adjustment for multiple comparisons
☐	☒	A full description of the statistical parameters including central tendency (e.g. means) or other basic estimates (e.g. regression coefficient) AND variation (e.g. standard deviation) or associated estimates of uncertainty (e.g. confidence intervals)
☐	☒	For null hypothesis testing, the test statistic (e.g. F, t, r) with confidence intervals, effect sizes, degrees of freedom and P value noted<br>*Give P values as exact values whenever suitable.*
☒	☐	For Bayesian analysis, information on the choice of priors and Markov chain Monte Carlo settings
☒	☐	For hierarchical and complex designs, identification of the appropriate level for tests and full reporting of outcomes
☐	☒	Estimates of effect sizes (e.g. Cohen's *d*, Pearson's *r*), indicating how they were calculated

Our web collection on statistics for biologists contains articles on many of the points above.

Software and code

Policy information about availability of computer code

Data collection	All behavioral tasks and recordings were controlled by custom written VC++ based software Version 16.7 (Blip; http://www.robilis.com/blip/). Data acquisition and output control was performed using National Instruments NI-PCIe 6353.
Data analysis	All behavioral and neural analysis as well as statistical testing was done using MATLAB 2017b. MRI localization with standard atlas was done using NMT toolbox (Version 1) https://afni.nimh.nih.gov/pub/dist/doc/htmldoc/nonhuman/macaque_tempatl/template_nmtv1.html

For manuscripts utilizing custom algorithms or software that are central to the research but not yet described in published literature, software must be made available to editors and reviewers. We strongly encourage code deposition in a community repository (e.g. GitHub). See the Nature Portfolio guidelines for submitting code & software for further information.

Data

Policy information about availability of data

All manuscripts must include a data availability statement. This statement should provide the following information, where applicable:

- Accession codes, unique identifiers, or web links for publicly available datasets
- A description of any restrictions on data availability
- For clinical datasets or third party data, please ensure that the statement adheres to our policy

All data needed to evaluate the conclusions in the paper are present in the paper and/or the Supplementary Materials. The neural data from vlPFC and SNr and the behavioral choice and free viewing data reported in this study are provided in the Supplementary Information/Source Data file.

March 2021

Field-specific reporting

Please select the one below that is the best fit for your research. If you are not sure, read the appropriate sections before making your selection.

☒ Life sciences ☐ Behavioural & social sciences ☐ Ecological, evolutionary & environmental sciences

For a reference copy of the document with all sections, see nature.com/documents/nr-reporting-summary-flat.pdf

Life sciences study design

All studies must disclose on these points even when the disclosure is negative.

Sample size	To calculate the sample size needed, we wanted to be able to detect changes of at about 0.1 (i.e., AUC > 0.6 or < 0.4) in mean population AUC with power 80% and significance level of 0.05. Our initial recording in each monkey showed a standard deviation of about 0.15-0.2 in AUC histogram across the population. Given these values and assuming a normal distribution for AUC, the total number of neurons to have significant results separately in each monkey, was found to be 18-32 neurons per animal per condition. We mostly match (Fig 3) or exceed this number (Fig 1) in our samples. http://powerandsamplesize.com/Calculators/Test-1-Mean/1-Sample-Equality.
Data exclusions	No data was excluded
Replication	Two monkeys and a large number of stimuli were used in this study to ensure replicability. Results of subjects are separately presented in the paper. We had no problems with replication.
Randomization	Allocation of stimuli to good and bad objects were random and the tasks run each day for each monkey was randomized.
Blinding	Obviously the subjects (monkeys) were blind to the conditions. Given the large number of stimuli their contingency was generally unknown to the experimenter. The analysis was not blind.

Reporting for specific materials, systems and methods

We require information from authors about some types of materials, experimental systems and methods used in many studies. Here, indicate whether each material, system or method listed is relevant to your study. If you are not sure if a list item applies to your research, read the appropriate section before selecting a response.

Materials & experimental systems

n/a	Involved in the study	
☒	☐	Antibodies
☒	☐	Eukaryotic cell lines
☒	☐	Palaeontology and archaeology
☐	☒	Animals and other organisms
☒	☐	Human research participants
☒	☐	Clinical data
☒	☐	Dual use research of concern

Methods

n/a	Involved in the study	
☒	☐	ChIP-seq
☒	☐	Flow cytometry
☐	☒	MRI-based neuroimaging

Animals and other organisms

Policy information about studies involving animals; ARRIVE guidelines recommended for reporting animal research

Laboratory animals	Two male adult rhesus monkeys (Macaca mulatta) were used in all tasks (monkeys B and R ages 7 and 10, respectively).
Wild animals	No wild animals were used in this study
Field-collected samples	No field-collected samples were used in this study.
Ethics oversight	All animal care and experimental procedures were approved by the National Eye Institute Animal Care and Use Committee and complied with the Public Health Service Policy on the humane care and use of laboratory animals.

Note that full information on the approval of the study protocol must also be provided in the manuscript.

Magnetic resonance imaging

Experimental design

Design type	no task - we only used structural MRI for determining the recording locations.
Design specifications	n/a
Behavioral performance measures	n/a

Acquisition

Imaging type(s)	structural, T1 and T2
Field strength	4.7 Tesla Bruker
Sequence & imaging parameters	1mm cube resolution T1 and T2 + Fieldmap and FLASH
Area of acquisition	Whole brain

Diffusion MRI ☐ Used ☒ Not used

Preprocessing

Preprocessing software	The structural analysis was performed in AFNI (AFNI_18.2.09) and ImageJ 2012-2015
Normalization	Mostly native space was used for daily navigation. However to verify atlas location the D99 atlas was used.
Normalization template	D99 atlas when needed
Noise and artifact removal	n/a
Volume censoring	n/a

Statistical modeling & inference

Model type and settings	n/a
Effect(s) tested	n/a

Specify type of analysis: ☐ Whole brain ☐ ROI-based ☐ Both

Statistic type for inference (See Eklund et al. 2016)	n/a
Correction	n/a

Models & analysis

n/a	Involved in the study
☒	☐ Functional and/or effective connectivity
☒	☐ Graph analysis
☒	☐ Multivariate modeling or predictive analysis
